# Zucchini Plants Alter Gene Expression and Emission of (*E*)-β-Caryophyllene Following *Aphis gossypii* Infestation

**DOI:** 10.3389/fpls.2020.592603

**Published:** 2021-01-08

**Authors:** Alessia Vitiello, Donata Molisso, Maria Cristina Digilio, Massimo Giorgini, Giandomenico Corrado, Toby J. A. Bruce, Nunzio D’Agostino, Rosa Rao

**Affiliations:** ^1^Department of Agricultural Sciences, University of Naples Federico II, Naples, Italy; ^2^Laboratory of Entomology, Wageningen University, Wageningen, Netherlands; ^3^Sede Secondaria di Portici, Istituto per la Protezione Sostenibile delle Piante, CNR, Portici, Italy; ^4^School of Life Sciences, Faculty of Natural Sciences, Keele University, Staffordshire, United Kingdom

**Keywords:** *Cucurbita pepo*, RNA-sequencing, transcriptome reprogramming, insect pests, zucchini–aphid interaction, phloem feeders, volatile organic compounds

## Abstract

Zucchini (*Cucurbita pepo* L.) is widely cultivated in temperate regions. One of the major production challenges is the damage caused by *Aphis gossypii* (Homoptera: Aphididae), a polyphagous aphid, which can negatively affect its host plant, both directly by feeding and indirectly by vectoring viruses. To gain insights into the transcriptome events that occur during the zucchini–aphid interaction and to understand the early-to-late defense response through gene expression profiles, we performed RNA-sequencing (RNA-Seq) on zucchini leaves challenged by *A. gossypii* (24, 48, and 96 h post-infestation; hpi). Data analysis indicated a complex and dynamic pattern of gene expression and a transient transcriptional reconfiguration that involved more than 700 differentially expressed genes (DEGs), including a large number of defense-related genes. The down-regulation of key genes of plant immunity, such as leucine-rich repeat (LRR) protein kinases, transcription factors, and genes associated with direct (*i.e.*, protease inhibitors, cysteine peptidases, etc.) and indirect (*i.e.*, terpene synthase) defense responses, suggests the aphid ability to manipulate plant immune responses. We also investigated the emission of volatile organic compounds (VOCs) from infested plants and observed a reduced emission of (*E*)-β-caryophyllene at 48 hpi, likely the result of aphid effectors, which reflects the down-regulation of two genes involved in the biosynthesis of terpenoids. We showed that (*E*)-β-caryophyllene emission was modified by the duration of plant infestation and by aphid density and that this molecule highly attracts *Aphidius colemani*, a parasitic wasp of *A. gossypii*. With our results we contributed to the identification of genes involved in cucurbit plant interactions with phloem feeders. Our findings may also help pave the way toward developing tolerant zucchini varieties and to identify molecules for sustainable management of harmful insect populations.

## Introduction

Aphids are major insect pests of crops that cause yield losses worldwide. These insects are phloem-feeders and belong to the Aphididae family, which comprises more than 4,300 species. They are mostly distributed in temperate regions where they colonize 25% of plant species (Dixon et al., 1987). Aphids deteriorate their host plants in different ways. As phloem-feeders they hijack the nutrients necessary to plant growth and reproduction for their own benefit, while as vectors they transmit several plant viruses causing a large number of diseases associated with huge production losses (Nault, 1997; Katis et al., 2007). Compared with chewing herbivorous insects, aphids are “stealthy” pests because they produce only very limited mechanical damage while feeding on phloem sap. However, due to their short generation time and consequent high populations, depletion of phloem sap can occur and seriously damage plants (Kuśnierczyk et al., 2008).

Insect pests cost billions of dollars in terms of crop losses and farmers face an ever-present threat of insecticide resistance due to overreliance on chemical control agents (Gordon and Waterhouse, 2007). Furthermore, extensive use of insecticides can cause tremendous damage to ecosystems, including the killing of beneficial insects (natural enemies and pollinators), substantial modification of soil communities, and gradual contamination of soil and water resources. Hence, there is an urgent need to develop alternative pest-control strategies, to allow a reduction in pesticide use. For all these reasons, the identification of genes and molecules able to contain harmful insect populations is a priority for modern agriculture (Le Mire et al., 2016).

Plants and herbivorous insects have been engaged in an arms race for hundreds of millions of years. Plant–aphid interactions are dynamic and subject to continual variation and change (Mello and Silva-Filho, 2002). Aphid attack is associated with a wide array of symptoms of different degrees of severity, which vary with plant and aphid species, and their combination (Miles and Peng, 1989; Goggin, 2007). In addition, during the feeding phase, aphids inject saliva that can manipulate host plant physiology by introducing effectors that modulate and suppress the defensive response of susceptible plants. Interaction with aphids results in both general host plant responses, such as preventing sieve tube plugging by molecular interactions between salivary proteins and calcium, and more specific molecular and biochemical responses (Will et al., 2013; Furch et al., 2015). As a consequence, the host molecular response is likely distinct for definite plant–aphid interactions. Aphid–plant interactions have been studied in several plant species but so far, no data are available for zucchini (*Cucurbita pepo* L.) plants.

In tomato plants, aphid infestation triggers after 48 h the up−regulation of a wide array of genes associated with salicylic acid (SA) biosynthesis or signaling (Coppola et al., 2018). For example, an increase in expression of the pathogenesis-related 1 gene (PR1) was observed in tomato plants susceptible to *Macrosyphum euphorbiae* (Coppola et al., 2013). Similarly, increased production of β-glucanase, PR1, and thaumatin-like proteins was observed in barley infested by *Rhopalosiphum padi* (Delp et al., 2009). One of the key regulatory elements in SA-dependent responses is the deoligomerization of the NPR1 (NON-EXPRESSOR OF PR1 GENE) protein into its active monomeric forms, which can interact with transcription factors (*e.g.*, TGA, WRKY) and activate PR gene expression and subsequent defense response (Verma et al., 2016). In addition, we previously demonstrated that when zucchini plants are treated with methyl salicylate, a volatile compound derived from SA, aphid dispersal behavior is similar to that in plants that experienced a previous infestation (Coppola et al., 2018). Overall, these findings suggest that SA plays an important role either in the activation of plant defense against aphids or aphid suppression of plant defense.

The expression of other defense genes is mediated by jasmonic acid (JA) and ethylene (ET).

The activation of the JA-pathway can substantially reduce aphid population (Ellis et al., 2002) although suppression of JA signaling in the tomato mutant *jai1* had no effect on *M. euphorbiae* population levels (Thompson and Goggin, 2006). The role of ET in plant defense is mainly mediated by the transcription factor EIN3 (ET INSENSITIVE3), which was suggested to induce the expression of the *ERF1* gene and mediate ET-signaling pathway (Solano et al., 1998). However, compared with chewing pests and mechanical wounding, aphids had a weak influence on JA- and ET-related gene expression. In general, SA antagonizes the JA-induced pathway during plant–aphid interactions, whereas ET can have both positive and negative effects to achieve tailored defense responses.

Zucchini belongs to the *Cucurbitaceae* family and ranks among the highest-valued vegetables worldwide. It is widely cultivated in temperate regions of the world where one of the main constraints to its cultivation is the damage imposed by *Aphis gossypii* (Homoptera: Aphididae), a polyphagous species known as cotton or melon aphid, which can both directly and indirectly negatively affects host plants.

To date, a few transcriptomic studies on the interaction between cucurbits and phloem-feeders have been published. Research performed on *A. gossypii*-susceptible and resistant accessions of melon (*Cucumis melo* L.) evidenced a stronger induction of the ET regulatory pathway in resistant melon, thus suggesting an important role of ET in *A. gossypii* resistance (Anstead et al., 2010). Data from the same study also support the ET-dependent activation of the JA pathway. JA-ET synergism was also observed in *Cucurbita moschata* (Duchesne) attacked by the silver leaf whitefly *Bemisia tabaci* (Gennadius) (van de Ven et al., 2000). Transcriptional reprogramming of cucumber plants (*Cucumis sativus* L.) infested by *A. gossypii* evidenced changes in the expression of genes likely associated with resistance to aphid-induced damage (Liang et al., 2015).

In this context, our study aimed at characterizing the early-to-late defense response of zucchini plant challenged by *A. gossypii* at three time points through RNA-sequencing (RNA-Seq)-based gene expression profiling.

To accomplish a more comprehensive understanding of the zucchini plant response, we investigated the kinetics of emission of volatile organic compounds (VOCs) from infested plants and identified links with the transcriptional reconfiguration. Finally, we studied the responses of aphid parasitoids to these VOCs.

## Materials and Methods

Details on the experimental design of the interaction between the aphid-susceptible *C. pepo* cultivar “San Pasquale” and *A. gossypii*, RNA isolation and sequencing, and transcriptome assembly and annotation are described in Vitiello et al. (2018).

Briefly, RNA-Seq was performed on leaves un-infested (control) and infested by five adult *A. gossypii*. More precisely, five aphids were transferred onto the adaxial surface of first and second leaves of plants individually enclosed in insect-proof cages (Supplementary Figure S1) and were left to feed for 24, 48, and 96 h, after which they were removed and leaves were sampled, pooled, and frozen in liquid nitrogen.

### Identification of Differentially Expressed Genes

A subset of 42,517 transcripts (*i.e.*, the longest transcript for each gene locus) was extracted from the assembled transcriptome (Transcriptome Shotgun Assembly accession GGKS01000000; Vitiello et al., 2018) and used as reference for read mapping. Bowtie2 (Langmead and Salzberg, 2012) was used to align reads (obtained after sequencing three biological replicates collected for both infested and control plants *per* time point) onto reference transcripts and was run in “end-to-end” mode using the “very-sensitive” option. Paired and unpaired reads were aligned by two independent runs. Read summarization was carried out using eXpress (Roberts and Pachter, 2013) with the “fr-stranded” option. Inter-sample normalization [trimmed mean of M-value (TMM)] was performed using edgeR (Robinson et al., 2010). TMM was applied on eXpress output files containing FKPM values (fragments per kilobase of exon model per million mapped fragments) after being rounded and filtered for low-abundance transcripts. Specifically, transcripts with FPKM values lower than 10 in more than three samples (biological replicates included) were filtered out. Furthermore, to evaluate homogeneity of the samples, Pearson’s correlation coefficient between biological replicates was calculated. EdgeR was also run for the call of differentially expressed genes (DEGs). Control and infested samples for each time point were compared in pairs. DEGs were identified by setting a false discovery rate (FDR) of 5% (FDR < 0.05) and a minimum log2 fold change (FC) of ±2.

Cluster analysis was performed using the Multiple Experiment Viewer (MeV, v.4.9.0) (Herrero et al., 2001). Transcripts were organized into clusters based on their expression profiles using the self-organizing tree algorithm (SOTA) and the Euclidean distance measure. Mercator sequence annotator 4 v2.0 (Schwacke et al., 2019) was used to assign each transcript to MapMan bins (functional categories/subcategories) (Thimm et al., 2004; Usadel et al., 2009). The resulting mapping file was imported in MapMan v3.6.0 in order to map DEGs onto MapMan bins for data visualization and pathway analysis.

### Quantitative Real-Time PCR (qRT-PCR)

Twelve genes with significant differences in RNA-Seq-based expression values for at least one of the three time points (i.e., 24, 48, and 96 hpi) were analyzed by qRT-PCR. Complementary DNA synthesis was performed using the same RNA samples used for RNA-sequencing and isolated from three biological replicates *per* time point from both infested and control plants. One μg of RNA was used as template for the SuperScript First-Strand cDNA Synthesis Kit (Invitrogen, Thermo Fisher Scientific, Wilmington, DE, United States) according to manufacturer’s instructions. The qRT-PCR mixture comprised of 5 μl QuantiFast SYBR Green PCR Master Mix (2x, Qiagen, Hilden, Germany), 0.4 μl of each primer (10 μM), 1 μl of cDNA (diluted 20-fold), and RNase-free water up to 10 μl. The reactions were performed on Corbett Rotor-Gene 6000 Real-Time PCR machine (Corbett Life Science/Qiagen) by the three-step method, which was initiated by 3 min at 95°C, then followed by 40 cycles of 95°C for 15 s, 60°C for 60 s, and 72°C for 20 s, and completed with a melting curve analysis program. Ct values were determined on three biological replicates. Relative expression levels of target genes were calculated using the 2^–ΔΔ*Ct*^ method (Livak and Schmittgen, 2001). The Elongation factor 1-alpha (HO702383) (Obrero et al., 2011) was used as the endogenous reference gene for the normalization of the expression levels of the target genes. Differences in relative quantities were analyzed by comparing ΔC_*t*_ values by a two-tailed *t*-test. Primer sequences and their main features are reported in Supplementary Table S1.

### Air Entrainment of Zucchini Plants

Headspace samples of VOCs were collected from un-infested and aphid-infested 4-week-old zucchini plants. Foliage of both un-infested and infested potted plants was placed in glass vessels (30 cm high × 15.5 cm internal diameter) with three collection holes at the top (one for inlet of the air and two for outlet), and closed at the bottom using two semi-circular aluminum plates that fitted around the plant stem. Headspace samples were trapped on a Porapak Q filter (50 mg, 50/80 mesh, Supelco Inc., Bellefonte, PA, United States) and Tenax TA filter (50 mg, 60/80 mesh, Supelco). Air, purified by passage through an activated charcoal filter, was pumped in at 600 ml/min and drawn out at 400 ml/min through the Porapak Q and Tenax TA tubes.

The first sampling was from plants that were infested with five adults for 48 h. Aphids were confined to the first real leaf, using a clip cage, and after 48 h were manually removed using a paintbrush. Infested and un-infested plants were then individually enclosed in glass vessels, excluding the infested leaf. VOCs were collected for a period of 24 h following removal of aphids. The time point was selected based on RNA-Seq results that showed the highest number of DEGs at 48 hpi. In the second sampling, whole plants were infested with 300 aphids (mixed ages) and were entrained for 7 days. Adsorbent tubes were changed at 24 h intervals for the first 4 days. Then, a 3-day collection was performed between the fourth and the seventh day, for a total of five samples collected *per* replicate. Seven replicates for both infested and un-infested plants were collected for each experiment. The collected samples were stored at −20°C before proceeding with downstream analyses.

### Gas Chromatography (GC) Analysis

Volatile samples collected from Porapak Q filter tubes were eluted twice with 250 μl of freshly distilled diethyl ether and 4 μl aliquots were analyzed on a gas chromatography (GC) (Agilent 7890A; Agilent, Edinburgh, United Kingdom) equipped with a cool on-column injector, flame ionization detector (FID), a non-polar HP-1 column (50 m × 0.32 mm × 0.52 μm film thickness, J & W Scientific) and a polar DB-WAX column (50 m × 0.32 mm × 0.52 μm film thickness). The initial GC oven temperature was 30°C for 0.5 min, increased to 150°C at a rate of 5°C/min, and then programmed at 10°C/min to 230°C for 27 min.

Tenax tubes were analyzed using a GC (Agilent 6890N) equipped with an integrated programmable injector (Optic 2, ATAS GL International, Eindhoven, Netherlands), a split/splitless injector, a non-polar HP-1 capillary column (50 m × 0.32 mm × 0.52 μm film thickness), and a FID detector. The GC initial oven temperature was maintained at 30°C for 1 min, programmed at 5°C/min to 150°C and held for 0.1 min, and then programmed at 10°C/min to 250°C for 20 min. The optic unit was programmed to start at 35°C and then rise to 250°C at 16°C/s. The carrier gas was hydrogen.

Quantification of (*E*)-β-caryophyllene was carried out on a non-polar HP-1 GC column, using a multiple point external standard method. A calibration curve (peak area vs concentration) was made using 0.1, 0.5, 1, 2, and 10 ng/μl concentrations of an authentic standard (Sigma Aldrich, Gillingham, United Kingdom) (*r*^2^ = 1). Statistical analysis was performed using a paired *t*-test (GenStat 2014, Seventeenth Edition, VSN International Ltd., Hemel Hampstead, United Kingdom).

### Coupled Gas Chromatography-Mass Spectrometry (GC/MS)

Headspace samples collected by elution form Porapak Q filter tubes were concentrated to ∼50 μl, and 4 μl aliquots were analyzed using a capillary GC column (50 m × 0.32 mm × 0.52 μm film thickness, HP-1, Agilent 6890N) fitted with a cool-on-column injector and coupled with a Micromass Autospec Ultima magnetic sector mass spectrometer. Ionization was performed by electron impact (70 eV, 220°C). Coupled GC/mass spectrometry (MS) analyses of Tenax filter tubes were performed on a Thermo Finnigan Mat95 XP magnetic mass spectrometer, equipped with a PTV unit (ATAS GL International, Eindhoven, Netherlands) and a Thermo Finnigan Trace GC, fitted with a non-polar HP-1 column (50 m × 0.32 mm × 0.52 μm film thickness). Ionization was by electronic impact (70 eV, 220°C). The oven temperature was maintained at 30°C for 5 min and then increased to 250°C at a rate of 5°C/min, for a run time of 70 min. The carrier gas was helium. Identification of compounds was made by comparing peak fragmentation profiles with those of authentic samples in the mass spectral database National Institute of Standards and Technology (NIST, version 2011) or by spectra interpretation. Initial identifications were confirmed by co-injection of the air entrainment sample with authentic standards on both non-polar HP-1 and polar DB-WAX GC columns, with peak enhancement indicating co-elution. Co-injection was performed for (*E*)-β-caryophyllene, humulene, nonanal, decanal, 6-methyl-5-hepten-2-one, and 3-octanone.

### Four-Arm Olfactometer Bioassays

Female *Aphidius colemani* parasitic wasps (purchased from Agralan, United Kingdom) and individual alate *A. gossypii* were introduced into a Perspex four-arm olfactometer (Pettersson, 1970) as described by Bruce et al. (2005) to determine their behavioral responses to synthetic (*E*)-β-caryophyllene (100 ng). A single insect was introduced into the central chamber of the apparatus and the time spent and number of entries into each arm was recorded using dedicated software (OLFA, Udine, Italy) over a 12 min period. Twelve observations were performed for each insect. The olfactometer was rotated of 90° every 3 min to reduce any directional bias. The mean time spent in and number of entries into treated and control arms were compared using a paired *t-*test (GenStat 2014).

## Results

### Changes in Gene Expression in Aphid-Infested Zucchini Plants

Transcriptome reprogramming in aphid-infested zucchini plants at 24, 48, and 96 hpi was analyzed to describe variation in gene expression following the establishment of a compatible interaction with *A. gossypii*.

The raw read count matrix was filtered to remove low-abundance genes and the resulting matrix was subjected to inter-sample normalization. Box plots describing the distribution of read counts before and after TMM normalization are shown in Supplementary Figure S2. Pearson’s correlation values between replicates ranged from 0.80 to 0.99 (Supplementary Figure S3).

**FIGURE 1:**
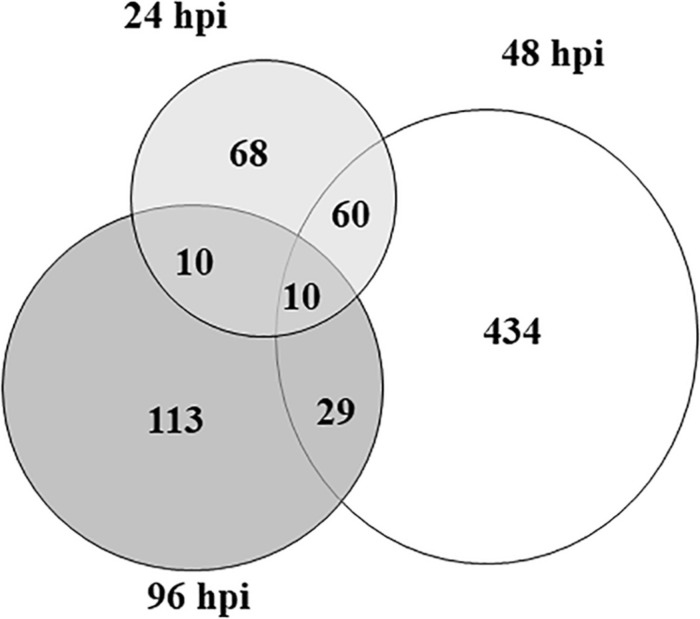
The area-proportional Venn diagram shows the number of overlapping and non-overlapping differentially expressed genes in zucchini “San Pasquale” challenged by *Aphis gossypii* vs control leaves at the three sampling times. At each time point, three biological replicates harvested from infested and control plants were compared. hpi, hours post-infestation.

By comparing treated vs control samples, 148 DEGs (109 up- and 39 down-regulated) were identified at 24 hpi, while the number of DEGs was 533 (390 up- and 143 down-regulated) and 163 (54 up- and 109 down-regulated) at 48 and 96 hpi, respectively (Figure 1). Clearly, DEG overlap among samples was moderate for all combinations, and only 10 transcripts were shared among the three time points (Table 1). The complete list of DEGs, including their expression profiles at the three time points, is provided in Supplementary Table S2.

**TABLE 1:** List of zucchini DEGs shared among three sampling times in response to *Aphis gossypii* feeding.

TC_ID	24 hpi		48 hpi		96 hpi		vs TAIR10		vs *C. pepo* gene models	
	Log FC	FDR	Log FC	FDR	Log FC	FDR	Sequence ID	Description	Gene ID	Description
CUCPE_ TC12321	2.561	0.007	2.911	0.001	2.545	0.012	AT1G24620.1	EF hand calcium-binding protein family	Cp4.1LG02g03370.1	Probablecalcium-binding CML29
CUCPE_ TC420	3.728	0.000	4.040	0.000	2.722	0.000	AT1G64720.1	Polyketidecyclase/ dehydrase and lipid transport superfamily protein	Cp4.1LG14g02150.1	Phosphatidylcholine transfer
CUCPE_ L14332_T_1	6.730	0.000	2.498	0.000	2.547	0.018	AT1G74630.1	Tetratricopeptide repeat (TPR)-like superfamily protein	Cp4.1LG20g00350.1	Pentatrico peptiderepeat-containing At1g74630
CUCPE_TC443	4.121	0.000	3.496	0.000	3.047	0.011	AT3G46010.2	Actindepolymerizingfactor1	Cp4.1LG20g08850.1	Actin-depolymerizingfactor 2
CUCPE_ L19052_T_1	2.235	0.002	2.174	0.001	−2.083	0.026	AT3G49470.1	Nascent polypeptide-associated complex subunit alpha-like protein 2	Cp4.1LG05g05740.1	Nascent polypeptide-associated complex subunit alpha 2
CUCPE_ TC20082	5.349	0.018	5.168	0.002	6.844	0.002	#N/A	#N/A	#N/A	#N/A
CUCPE_ TC17141	5.659	0.001	10.890	0.000	7.697	0.000	#N/A	#N/A	#N/A	#N/A
CUCPE_ L12655_T_1	6.331	0.001	7.525	0.000	4.948	0.034	#N/A	#N/A	#N/A	#N/A
CUCPE_ L16873_T_1	6.561	0.000	10.062	0.000	5.138	0.028	#N/A	#N/A	#N/A	#N/A
CUCPE_ L1594_T_1	−4.419	0.000	−3.213	0.001	5.004	0.000	AT5G42070.1	Unknown protein	Cp4.1LG01g00520.1	#N/A

*For each transcript at different time points [namely, 24, 48, and 96 h post-infestation (hpi)], it is reported the expression level estimate (Log2 fold-change) and the statistical significance value as calculate by edgeR (FDR). Functional annotation is obtained using the following resources: *A. thaliana* proteins (TAIR 10, https://www.arabidopsis.org/); *C. pepo* genome (v4.1, https://bioinf.comav.upv.es/downloads/zucchini/genome_v4.1/). #N/A, not annotated.*

The qRT-PCR-based expression profiles of 12 genes were compared with RNA-Seq-based gene expression values at each time point (Supplementary Figure S4).

### Timing and Dynamics of the Zucchini Response to Aphids

**FIGURE 2:**
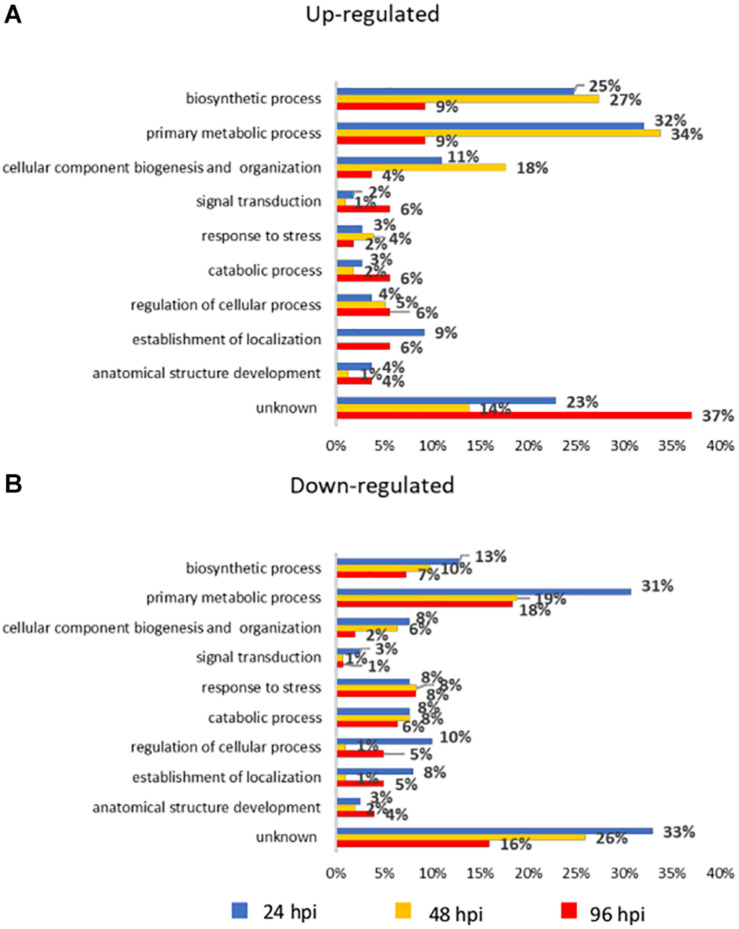
Bar chart describing gene ontology (GO) annotation of differentially expressed genes (DEGs). Up-regulated **(A)** and down-regulated **(B)** genes were assigned to GO terms in the “biological process” domain level 3. The *x*-axis indicates the percentage of DEGs in each category out of the total number of DEGs at each time point. Bars with the same color code refer to the same time point. hpi, hours post-infestation.

An overview of the major biological processes influenced by *A. gossypii* feeding activity on zucchini plants were investigated by associating DEGs to gene ontology (GO) terms. These were distributed in 10 categories (Figure 2) and their distribution across the three time points reveals that transcriptome reconfiguration involved a broad range of biological processes.

**FIGURE 3:**
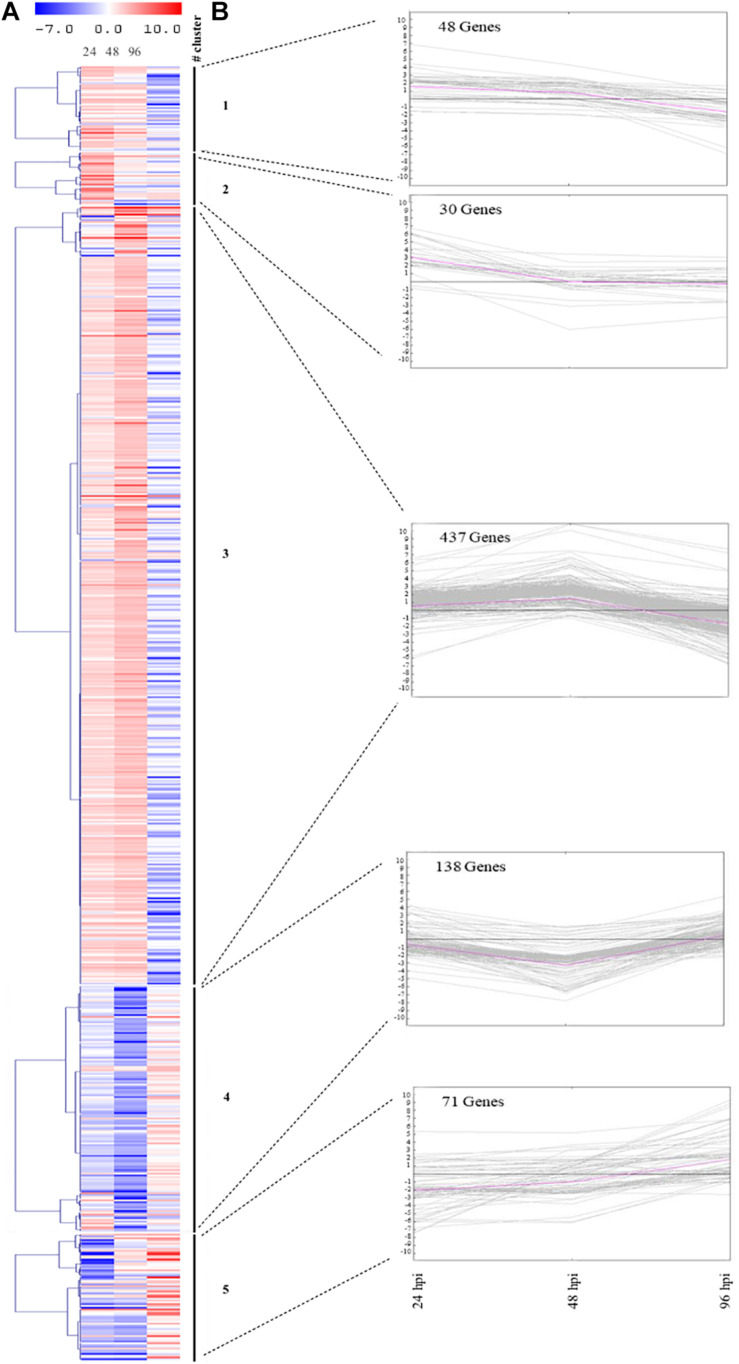
Gene expression changes in zucchini leaves after infestation with *Aphis gossypii*. **(A)** Heatmap of the 724 DEGs in at least one out of three comparisons (24, 48, and 96 hpi). **(B)** SOTA clustering of genes with comparable expression profiles throughout the interaction period (24, 48, and 96 hpi). The number of genes assigned to each cluster is indicated. The pink line refers to the cluster centroid. On the *y*-axis is the fold change of the gene expression level. hpi, hours post-infestation.

Furthermore, the heatmap reflects the dynamics of gene expression in zucchini plants challenged by aphids (Figure 3A). SOTA algorithm analysis allowed grouping DEGs into five clusters based on the similarity of their gene expression profiles (Figure 3B). Cluster 3 includes the highest number of genes (437), the majority of which (372) were up-regulated at 48 hpi; the 20% of genes within the cluster showed down-regulation at 96 hpi. Conversely, the 86% of genes belonging to cluster 4 (138) were strongly down-regulated at 48 hpi. Clusters 1 and 2 include the lowest number of genes (48 and 30, respectively). For cluster 2, up-regulation of genes at 24 hpi was observed, followed by decrease in expression levels in the remaining time points. On the other hand, genes in cluster 1 were up-regulated or marginally affected by infestation at 24 and 48 hpi, but were down-regulated at the last time point. Finally, cluster 5 includes 71 genes, which were severely down-regulated at 24 and 48 hpi, followed by an increase in gene expression at 96 hpi (Figure 3B).

**FIGURE 4:**
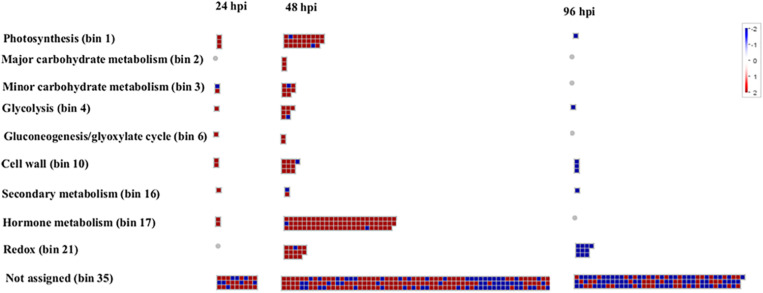
Organization of zucchini differentially expressed genes (DEGs) in functional categories (bins) according to the MapMan ontology across the interaction period (24, 48, and 96 h post-infestation). Genes significantly up-regulated and down-regulated in *Aphis gossypi* challenged vs control plants are indicated in red and blue, respectively. The color set scale is on top right. hpi, hours post-infestation.

An overview of the distribution of DEGs at 24, 48, and 96 hpi, based on MapMan ontology, is provided in Figure 4. In order to allow the reader to easily move through the data, each DEG was associated with its corresponding MapMan bin code and name (Supplementary Table S2). Figure 4 shows the wide-ranging impact of *A. gossypii* infestation on the transcriptional regulation of zucchini genes associated with the most populated bins, the first of which is bin 35 (not assigned), which also includes several genes associated with general defense response.

Zucchini genes whose expression is modulated following interaction with the aphid were grouped according to their functional annotation (Supplementary Table S2).

### Zucchini Transcriptional Response to Aphids

#### Signal Transduction and Defense Responses

**FIGURE 5:**
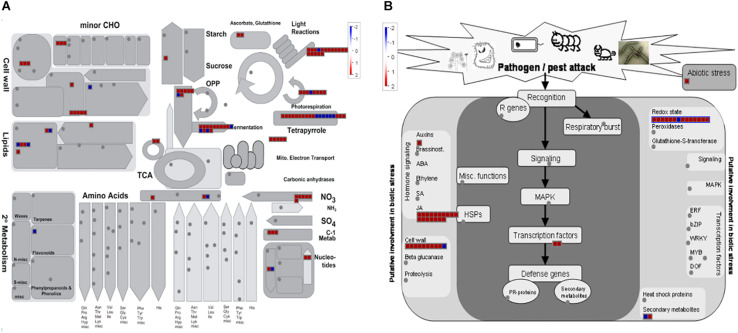
MapMan overview of zucchini differentially expressed genes at 48 h post-infestation by *Aphis gossypi* related to metabolism **(A)** and biotic stress **(B)**. Red and blue boxes correspond to up-regulated and down-regulated genes, respectively. The gray circles represent not differentially expressed genes.

Following aphid attack several genes involved in oxidative stress response, defense molecule biosynthesis, signaling pathways, and in response to biotic and abiotic stress were regulated at the transcriptional level. A total of 30 genes associated with oxidative stress were differentially expressed, indicating that the cell redox state was altered. Up-regulation of genes encoding for ROS-detoxifying enzymes occurred mainly at 48 hpi (Figure 5B). ROS cellular damage could result in lipid membrane oxidation and accumulation of toxic compounds (*e.g.*, reactive aldehydes). Two aldehyde dehydrogenases (CUCPE_L1947_T_4, CUCPE_L739_T_1) and one aldo/keto reductase (CUCPE_TC17159) were up-regulated at 48 and 96 hpi, respectively (Figure 5B and Supplementary Figure S5B). Six genes annotated as peroxiredoxins were up-regulated at 24 (CUCPE_TC11397, CUCPE_TC18555, CUCPE_L18317_T_5, CUCPE_TC12356, CUCPE_TC10421, CUCPE_TC12357) and 48 hpi (CUCPE_TC12356) (Figure 5B and Supplementary Figure S6B). In addition, three genes (CUCPE_L750_T_2, CUCPE_L2096_T_2, CUCPE_L673_T_12) encoding for members of the cytochrome P450 family were deregulated. At 48 hpi, genes encoding for a Cu/Zn superoxide dismutase (CUCPE_TC17415) (Figure 5B), a peroxidase (CUCPE_TC5181) and three glutaredoxins (CUCPE_L17883_T_2, CUCPE_TC1459, CUCPE_L1197_T_1) were up-regulated. Conversely, two glutathione S-transferases (CUCPE_TC18969, CUCPE_L26560_T_1) and five peroxidases (CUCPE_TC13568, CUCPE_L17764_T_4, CUCPE_TC859, CUCPE_L728_T_3, CUCPE_TC18638) were down-regulated at the same time point. After 24 and 48 hpi, six members (CUCPE_TC250, CUCPE_TC251, CUCPE_L190_T_15, CUCPE_TC249, CUCPE_L3725_T_3, CUCPE_TC13244) of the protochlorophyllide oxidoreductase (POR) enzyme family were strongly up-regulated.

Five genes encoding for calcium-binding proteins (CUCPE_TC12321, CUCPE_TC20529, CUCPE_L9238_T_1, CUCPE_L17253_T_1, CUCPE_L20074_T_1) and a calmodulin-like protein (CUCPE_L20750_T_1) were up-regulated at different time points. A gene (CUCPE_TC12321) encoding for a calcium-binding EF hand family protein was up-regulated during the whole time course, while a calmodulin-like encoding gene (CUCPE_L7 28_T_3) was down-regulated at 48 hpi.

Moreover, aphid feeding altered expression of transcripts encoding for proteins with kinase and/or receptor activity, which were both up-regulated (CUCPE_TC22434, CUCPE_TC3327) and down-regulated (CUCPE_L14082_T_1, CUCPE_L348_T_23) at 48 hpi. At 96 hpi, two genes annotated as leucine-rich repeat (LRR) receptor-like protein kinase (CUCPE_L1464_T_4, CUCPE_L16201_T_1) were up regulated, while four genes encoding for enzymes with kinase activity (CUCPE_TC19413, CUCPE_TC17500, CUCPE_TC22434, CUCPE_TC83) were down-regulated.

The transcriptional activity of several genes involved in abiotic stress response was modulated during the three time points, including five genes related to heat shock (CUCPE_L395_T_22, CUCPE_L6339_T_5, CUCPE_L320_T_3, CUCPE_L12331_T_1, CUCPE_L638_T_2), six chaperone proteins (CUCPE_TC16054, CUCPE_TC1321, CUCPE_L1696_T_2, CUCPE_TC2105, CUCPE_L568_T_4, CUCPE_TC555), and two ubiquitin ligase proteins (CUCPE_TC4482, CUCPE_TC1752).

#### Transcription-Related Genes

A total of 28 DEGs associated with transcriptional regulation were identified. Three genes (CUCPE_TC20338, CUCPE_TC11181, CUCPE_L14332_T_1) annotated as pentatricopeptide repeat-containing proteins (PPR) were up-regulated during the entire time frame, while a different member of the same family (CUCPE_L15434_T_1) was down-regulated at 24 hpi.

Four genes encoding for BTB/POZ and TAZ domain-containing proteins, which mediate transcriptional regulation in response to Ca^++^, H_2_O_2_, and SA were down-regulated at 24 (CUCPE_TC21759) and 48 hpi (CUCPE_TC1881, CUCPE_L1540_T_10, CUCPE_TC21761, CUCPE_TC21759). In addition, genes involved in transcriptional regulation associated with plant stress response were both up- and down-regulated. Specifically, at 48 hpi a gene encoding for a NAC domain-containing protein (CUCPE_L9153_T_1) was up-regulated, while five members of the same family (CUCPE_TC12669, CUCPE_TC19797, CUCPE_L11075_T_1, CUCPE_L20277_T_2, CUCPE_TC19313) and two genes encoding for MYB-like transcription factors (CUCPE_TC21085, CUCPE_TC17820) were down-regulated. Finally, two members of the GATA transcription factors (CUCPE_TC6210, CUCPE_L8119_T_1), which are mostly implicated in light-dependent gene regulation, were down-regulated at 96 hpi.

#### Primary Metabolic Process

Zucchini plants response to *A. gossypii* infestation included regulation of primary metabolism. Indeed, genes associated with carbohydrates, amino acid, and lipid metabolism were regulated at transcriptional level (Figure 5A and Supplementary Figures S5A, S6A). Transcripts related to sugar metabolism were mainly up-regulated at 24 and 48 hpi as well as genes involved in glycolysis and TCA cycle. A gene (CUCPE_L9559_T_11) encoding for a ß-xylosidase and involved in the hydrolysis of polysaccharides was up-regulated at 48 hpi, but down-regulated at 96 hpi. The same expression pattern was recorded for two genes encoding for ß-galactosidases (CUCPE_TC2939, CUCPE_L11418_T_3).

The expression of several genes involved in lipid metabolism was altered. Among them, we observed at 24 and 48 hpi the up-regulation of four genes encoding for acyl carrier proteins (CUCPE_TC18933, CUCPE_TC18931, CUCPE_TC18932, CUCPE_L6532_T_3). At 48 hpi, two genes (CUCPE_TC10339, CUCPE_TC10336) encoding for the phosphomethylethanolamine *N*-methyltransferase enzymes (PEAMT; EC 2.1.1.103), which catalyze the key step in choline (Cho) biosynthesis, and two GDSL esterase/lipase 1 enzymes (CUCPE_TC15023, CUCPE_L26246_T_1) were deregulated. Finally, genes encoding for different classes of peptidases (CUCPE_TC9010, CUCPE_TC2897, CUCPE_L19182_T_1, CUCPE_TC20622, CUCPE_TC21618) were down-regulated at 96 hpi.

#### Secondary Metabolism Related Genes

Genes associated with secondary metabolism showed significant differential expression (Figure 5B and Supplementary Figures S5B, S6B). Genes involved in vitamin metabolism (CUCPE_L10047_T_1, CUCPE_TC166, CUCPE_TC12656, CUCPE_TC166) were up-regulated at 24 hpi and two of them (CUCPE_L10047_T_1, CUCPE_TC12656) were also up-regulated at 48 hpi. At 96 hpi, a transcript encoding for thiamine thiazole synthase (CUCPE_TC137), involved in the biosynthesis of the thiamine (vitamin B1) precursor thiazole, was up-regulated.

A 4-coumarate:CoA ligase (CUCPE_TC20042, 4CL: EC 6.2.1.12) was up-regulated at 24 and 48 hpi, while transcriptional activity of genes involved in non-mevalonate pathway (MEP) were affected by aphid feeding. Specifically, 4-hydroxy-3-methylbut-2-enyldiphosphatereductase (CUCPE_L536_T_4) was down-regulated at 24 hpi while a 2-C-methyl-D-erythritol 4-phosphate cytidylyltransferase (CUCPE_TC12799) was up-regulated at 48 hpi.

Among genes negatively affected by *A. gossypii* infestation, we found a gene encoding for a member of the RmlC-like cupins superfamily (CUCPE_TC9431), associated with the isopentenyldiphosphate biosynthetic process (Chang et al., 2013), down-regulated 48 hpi and a transcript encoding for a terpene synthase (CUCPE_L25896_T_1), strongly down-regulated at 96 hpi. Three genes encoding for polyketidecylcases/dehydrases (CUCPE_TC420, CUCPE_TC421, CUCPE_TC419), which are involved in the synthesis of CUCPE_TC238 polyketides, a class of compounds with antimicrobial and immune-suppressive properties, were up-regulated during the whole time course. Furthermore, two genes associated with xanthophyll biosynthesis (CUCPE_TC248, CUCPE_TC238) were up-regulated at 48 hpi.

#### Anatomical Structure Development

Pathogens and herbivorous insects affect the expression of genes related to the anatomical structure of the cell. Several genes active in cell wall metabolism and remodeling were differentially expressed after *A. gossypii* infestation. Eighteen transcripts were up-regulated at 24 and 48 hpi, whereas all genes within this functional class were down-regulated at 96 hpi (Figure 5B and Supplementary Figures S5B, S6B). We also observed the up-regulation of seven genes encoding for extension proteins (CUCPE_TC17105, CUCPE_L540_T_7, CUCPE_TC20954, CUCPE_TC17106, CUCPE_TC20256, CUCPE_TC635, CUCPE_L13037_T_3) and of four cellulose synthase enzymes (CUCPE_L320_T_3, CUCPE_TC1626, CUCPE_TC1625, CUCPE_TC1222). In addition, seven genes encoding for arabinogalactan (AGP) proteins (CUCPE_TC19819, CUCPE_TC963, CUCPE_L11717_T_1, CUCPE_TC22452, CUCPE_TC7427, CUCPE_L18480_T_1, CUCPE_TC12683), the most functionally important and abundant proteins of plant cell wall, were up-regulated at 48 hpi. Conversely, genes encoding for enzymes involved in cell wall degradation (CUCPE_TC10408, CUCPE_TC606, CUCPE_TC18166) were down-regulated at 96 hpi.

#### Transport and Cell Maintenance

Genes involved in water, ion, heavy metal, and metabolite transport were strongly affected by aphid infestation. Five genes (CUCPE_TC456, CUCPE_TC452, CUCPE_TC16518, CUCPE_TC16696, CUCPE_TC12901) encoding for aquaporin plasma membrane intrinsic proteins (PIPs), which have an important role in controlling membrane water permeability, were up-regulated at 48 hpi. Also, two genes (CUCPE_L23657_T_1, CUCPE_L16073_T_1) encoding for mitochondrial component of the TOM (translocase of outer membrane) receptor complex, were up-regulated at 48 hpi. Similar expression pattern was observed for genes associated annotated as copper (CUCPE_L9583_T_1), magnesium (CUCPE_TC17148), and ABC-2 type (CUCPE_L13300_T_1) transporter. After 96 hpi a member of the HMA family (CUCPE_L13518_T_1), putative cadmium/zinc-transporting ATPase HMA4, was up-regulated. At the same time point, one heavy metal-associated isoprenylated plant protein 26 (HIPP26) (CUCPE_TC9026) involved in heavy metal homeostasis and detoxification mechanisms was down-regulated.

Twenty-one transcripts involved in cell maintenance showed differential expression following aphid infestation. This category grouped genes coding for proteins implicated in cell cycle, cellular component organization, and cell differentiation. One gene (CUCPE_TC443) within the category, encoding for an actin depolymerizing factor 2, was up-regulated during the whole time frame. Genes involved in cytoskeleton organization and intracellular movement were also recorded as differentially expressed. For example, a transcript encoding for a kinesin-like protein (CUCPE_L1772_T_6), important in intracellular transport, mitosis, and meiosis, was up-regulated at 24 hpi. Finally, genes involved in cell cycle and nucleotide and nucleic acid metabolism were identified. Two cyclin-dependent kinases (CUCPE_TC11460, CUCPE_L10663_T_1) were up-regulated at 48 hpi, and two tubulin proteins (CUCPE_TC11388, CUCPE_L674_T_3) were down-regulated at 96 hpi. A total of six genes (CUCPE_TC16568, CUCPE_L18787_T_2, CUCPE_L2485_T_1, CUCPE_TC3933, CUCPE_L947_T_1, CUCPE_L517_T_1) involved in chromatin structure (three histones H2A and histone H2B, H3, and H4) were up-regulated at 48 hpi (Figure 5A).

#### Phytohormonal-Related Genes

*Aphis gossypii* feeding affected genes related to SA- and JA-signaling pathways (Figure 5 and Supplementary Figures S5, S6).

Up-regulation of (CUCPE_TC1380) ICS1 was observed at 24 and 48 hpi. Seven genes encoding for PR proteins were up-regulated both at 48 (CUCPE_TC9646, CUCPE_TC14419, CUCPE_L3318_T_1, CUCPE_L17539_T_4, CUCPE_TC10497, CUCPE_TC2531) and at 96 hpi (CUCPE_L7720_T_1). A gene annotated as NIM1-interacting 1 (NIMIN1, CUCPE_L11848_T_1), a regulator of the systemic acquired resistance, was down-regulated at 48 hpi. Among SA-related genes, members of the nudix hydrolase protein family were up-regulated at 24 hpi (CUCPE_TC17968) and down-regulated at 48 hpi (CUCPE_L17789_T_1, CUCPE_TC20679). JA/ET-related and wounding-related genes were mainly down-regulated during the whole time frame. At 48 hpi, the ET-responsive transcription factor AP2-7 (AP2/ERF, CUCPE_TC228) was up-regulated. Additionally, at 48 hpi, six members of the bHLH transcription factor family were both up- (CUCPE_L17224_T_1, CUCPE_TC18025, CUCPE_TC373, CUCPE_TC1799) and down-regulated (CUCPE_L20235_T_5, CUCPE_TC3339).

#### Volatile Organic Compounds Emitted by Infested Plants

**FIGURE 6:**
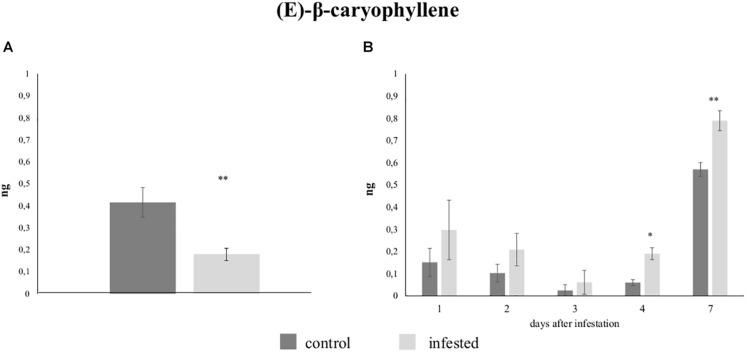
Quantitative differences in the emission of (*E*)-β-caryophyllene by control and infested zucchini leaves with **(A)** five *Aphis gossypii* adults after 48 h and with **(B)** 300 *A. gossypii* adults up to 7 days. Each bar shows the mean quantity expressed in ng (±SE) collected from seven replicates. Data were analyzed using a paired *t*-test, *P < 0.05; **P < 0.01.

Gas chromatography analysis revealed that only a few VOCs were released from leaves of both control plants and plants infested with five aphids. To understand if the low level of volatile production observed from infested plants was due to aphid density, we increased the number of aphids to 300 and increased the infestation time to 7 days. More compounds were detected although differences between control and infested plants were still not apparent (Supplementary Figure S7). Then, we collected VOCs on Tenax filters to see if this matrix could provide a higher resolution analysis. Again, the profile of VOCs emitted by infested plants was similar the profile from un-infested plants (Table 2). However, significant differences in the emission of (*E*)-β-caryophyllene were observed. The identity of this compound was confirmed through co-injection experiments with the chemical standard as shown in Supplementary Figure S8. (*E*)-β-caryophyllene emission was significantly reduced in plants infested for 48 h (0.18 ± 0.07 ng) when compared with un-infested plants (0.41 ± 0.17 ng; *p* = 0.003) (Figure 6A) while a significant increase was observed in zucchini plants infested with 300 aphids for 4 and 7 days (Figure 6B). Specifically, the amount of (*E*)-β-caryophyllene collected from leaves after 4 days of infestation (0.19 ± 0.06 ng) was greater than that from control plants (0.06 ± 0.03 ng; *P* = 0.041). Furthermore, the (*E*)-β-caryophyllene amount from leaves after 7 days of infestation (0.79 ± 0.1 ng) was significantly higher than that of un-damaged control plants (0.57 ± 0.07 ng; *P* = 0.013).

**TABLE 2:** Names and Kovats index (KI) of volatile organic compounds collected from *Aphis gossypii*-infested and un-infested zucchini plants.

# Peak	Compound ID	ExpKI*	KI**
1	Heptanal	883	883
2	Benzaldehyde	930	933
3	6-Methyl-5-hepten-2-one	967	966
4	3-Octanone	971	968
5	Acetophenone	1035	1040
6	Nonanal	1084	1084
7	Undecene	1090	1100
8	Decanal	1187	1186
9	(*E*)-β-caryophyllene	1425	1432
10	E-beta-farnesene	1448	1450
11	Humulene	1458	1465
12	Beta-selinene	1488	1489

Compounds here listed are obtained combining the volatiles identified in control and infested samples in both treatment conditions (5 and 300 *A. gossypii*). *Experimental KI. **Kovarts index.

#### Behavioral Response of Aphids and Parasitoids to Synthetic (*E*)-β-Caryophyllene

**FIGURE 7:**
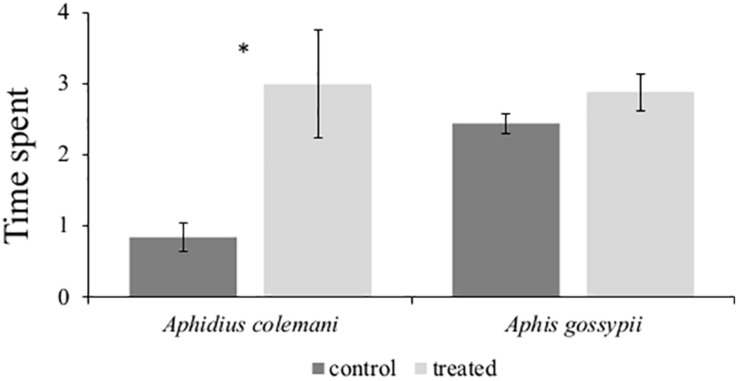
Behavioral responses of winged *Aphis gossypii* and parasitoid females *Aphidius colemani* in four-arm olfactometer (*n* = 12) to synthetic (*E*)-β-caryophyllene. Data are expressed as the mean (±SE) time (min) spent in treatment and control arms and were analyzed using a paired *t*-test. *P < 0.05.

In order to evaluate the behavioral responses of *A. gossypii* and the parasitic wasp *A. colemani* in the presence of (*E*)-β-caryophyllene, we performed a four-arm olfactometer bioassay. No differences were found between the time spent by aphids in the treated region of the olfactometer (2.88 ± 0.26 min) and in the control region (2.44 ± 0.14 min; *P* = 0.119). In contrast, *A. colemani* parasitoid females were remarkably attracted by (*E*)-β-caryophyllene, spending a significantly longer period of time in the treated (2.99 ± 0.86 min) than in the control sections (0.83 ± 0.20 min; *P* = 0.019) (Figure 7).

## Discussion

Core responses of plants to pests likely result from cross-talk between hormone-related pathways (Spoel and Dong, 2008; Pieterse et al., 2009; Nguyen et al., 2016), such as JA, SA, ET, ABA, and/or from signaling mediated by ROS (Torres et al., 2006), calcium (Dodd et al., 2010), and phosphorylation cascades (Asai et al., 2002). Transcription factors regulate transmission of these signals with consequent activation or repression of genes involved in immune responses and metabolic processes. Since most plant immune responses are under transcriptional control, transcriptome profiling is an effective tool to monitor the activation or suppression of specific regulatory and metabolic pathways during plant–pest interactions.

Our results describe the timing and dynamics of the zucchini plant responses to aphids and demonstrate that the interaction triggered a deep transcriptome reprogramming that involved more than 700 DEGs (Figures 1, 4 and Supplementary Table S2). We used the zucchini transcriptome described by Vitiello et al. (2018) as it was primarily established to be used as reference for the identification of DEGs. However, all transcripts were mapped onto the available *C. pepo* genome (see Supplementary Table S2) to maintain a link with the zucchini genome released into the public domain (Montero-Pau et al., 2018) and to facilitate comparison and annotation transfer.

### Zucchini Plants Activate a Marked and Rapid Transcriptome Reprogramming

Zucchini plant gene expression changed markedly during the infestation, promoting deep changes in biosynthetic and metabolic processes at all the time points. These results suggest that zucchini plant responses to aphid attack converge toward the transient production of compounds associated with plant defense along with a switch from development to defense. However, as this is a compatible interaction, some of the changes observed may be caused by aphid effectors and involve suppression of defenses by the aphid.

The number of DEGs increased significantly from 24 to 48 hpi and declined at 96 hpi (Figures 1, 4). Similar gene expression patterns were described in tomato plants attacked by *M. euphorbiae* (Coppola et al., 2013). In the first two time points, a significant increase in the number of up-regulated genes occurred, which peaked at 48 hpi, while the majority of DEGs were down-regulated at 96 hpi (Figure 4). This trend is likely the consequence of plant reactions that progressively mount the appropriate defenses during the initial steps of the perceived damage. Later, the attacked plant needs to balance the induction of defenses with growth and also to counteract the aphid’s ability to interfere with plant defense mechanisms (Mutti et al., 2008; Nalam et al., 2018).

Up-regulated transcripts common to the three time points (Table 1) are all associated with plant molecular responses to stresses. Specifically, CUCPE_TC12321 encodes for an EF hand calcium-binding protein. The EF-hand motif is the most common calcium-binding motif found in proteins. These proteins perceive changes in Ca^++^ concentrations and translate them into appropriate molecular and biochemical responses (Zhang et al., 2014 and the references therein). Similarly, CUCPE_TC420 encodes for a polyketidecyclase/dehydrase, which is involved in plant defense (Satheesh et al., 2014), while CUCPE_TC443, encoding for an actin-depolymerizing factor family protein that alters actin dynamics and mediates its depolymerization, was associated with biotic and abiotic stress responses in several plants and plays a role in the activation of resistance against biotic stresses in Arabidopsis (Tian et al., 2009; Inada, 2017). CUCPE_L14332_T_1 encodes for a protein belonging to the PPR family, which has a range of essential functions in RNA-editing events within mitochondria and chloroplasts also playing a key role in plant stress responses and development (Sharma and Pandey, 2016). Finally, CUCPE_L19052_T_1 encodes for a NACα protein (nascent polypeptide-associated complex subunit alpha-like protein 2) that usually binds to the newly synthesized nascent polypeptides as they emerge from the ribosome, showing chaperone function. NAC complex plays a role in response to biotic factors, and it was shown to be involved in hypersensitive response to pathogens in hot pepper (Huh et al., 2012). The up-regulation of these genes during the interaction suggests that they may have an important role in the adaptation of zucchini plant to the stress condition.

Plants perceive aphids through their molecular patterns that trigger adaptive plant immune responses. Plant responses, in attempt to neutralize aphid damage, can include the activation of metabolic shifts that produce *de novo* compounds or boost production of constitutive compounds able to reduce aphid growth and vitality (Züst and Agrawal, 2016; Coppola et al., 2019). For example, in our study, the up-regulation of CUCPE_TC20042 encoding for a 4-coumarate:CoA ligase, involved in the synthesis of flavonoids and lignin (Lee et al., 1997), modifies the flow in the phenylpropanoid metabolic pathway known to be involved in plant defense (Dixon et al., 2002). In addition, the up-regulation of a receptor-like kinase (CUCPE_TC22434) at 48 hpi is consistent with this scenario. At the same time point, we observed the up-regulation of a transcript related to phosphatases (CUCPE_TC3327), a family of proteins operating in a coordinated manner with the protein kinases, likely involved in keeping the defense systems in balance (Hong et al., 2017).

Several plant immune receptors hold a LRR protein motif that recognizes pathogen or herbivore effectors and activates the first line of defense (Wang et al., 2018). The LRR protein motif can also regulate immune receptor activity by engaging in intra-molecular interactions with other domains. Interestingly, a member of the LRR protein family (CUCPE_L16201_T_1) is strongly up-regulated only at 96 hpi. The late up-regulation of this transcript is likely associated with the susceptibility of the zucchini cultivar “San Pasquale” to aphids. In fact, it was previously shown that the early up-regulation of members of the LRR family protein is associated with increased resistance to biotic stresses (Komjanc et al., 1999; Sakamoto et al., 1999; Yan et al., 2018), while the absence of a functional LRR domain disrupts immunity (Borrelli et al., 2018). In addition, a remarkable difference between susceptible and resistant plants is in the timing with which they activate defense responses (Niu et al., 2018).

The down-regulation of many transcripts associated with oxidative stress (Supplementary Table S2) suggests that infested plants have an altered redox status. In addition, the down-regulation of transcripts encoding enzymes with oxidase activity or involved in ascorbic acid metabolic processes at 96 hpi may indicate a specific plant defense reaction against aphids as the reduction of these activities decreases the nutritional quality of the leaves and likely impairs aphid feeding (Rasool et al., 2017; Nalam et al., 2018). Therefore, the up-regulation of transcripts encoding for these enzymes, as well as for ROS-detoxifying enzymes, at 24 and 48 hpi indicates that the initial steps of zucchini response to aphids infestation are characterized by an increased oxidative stress, consistently with data observed in other plant species (Moloi and van der Westhuizen, 2006; Kerchev et al., 2012a,b; Liang et al., 2015; Borowiak-Sobkowiak et al., 2016), while their down-regulation occurring at 96 hpi might aim to reduce aphid growth (Nalam et al., 2018). These aspects should be better explored in future work.

In addition to local reactions, aphid infestation induces a systemic effect that modulates the expression of several plant genes, including those involved in cell wall modification, water transport, and vitamin biosynthesis (Divol et al., 2005). Our results are in line with those observations, as we observed the up-regulation of genes related to LRR extensin-like protein and other proteins involved in cell wall development, as well as in water transport (aquaporin) and vitamin biosynthesis (thiamine thiazole synthase, thiamine *C* phosphomethylpyrimidine synthase).

Pentatricopeptide repeat-containing proteins regulate gene expression at the RNA level. Their combined action largely affects several biological plant functions, including photosynthesis, development, regulation of the homeostasis of reactive oxygen species, and plant defense (Kishimoto et al., 2005; Laluk et al., 2011). The strong down-regulation of a PPR gene (CUCPE_L15434_T_1) observed in the initial step of the infestation (24 hpi) is likely associated with the required switch from growth vs defense to balance costs imposed by aphid attack. In contrast, the up-regulation observed at the later time points may be associated with plant defense reaction, as well as with the regulation of ROS. As a consequence of ROS production, lipid membranes might break with the accumulation of toxic compounds (*e.g.*, reactive aldehydes). The up-regulation of genes encoding for enzymes active in the detoxification mechanism of aldehydes (aldehyde dehydrogenases and aldo/ketoreductases) is consistent with the altered redox state of zucchini infested plants (Kishimoto et al., 2005; Laluk et al., 2011; Kim and Hwang, 2015; Sengupta et al., 2015). POR is an essential enzyme that catalyzes the photoreduction of protochlorophyllide to chlorophyllide, which is ultimately converted into chlorophyll in developing leaves (Garrone et al., 2015). However, this enzyme is also involved in oxidative stress, reducing plasma membrane damage in Arabidopsis and its over-expression was also associated with resistance to biotic stress (Tripathy, 2009; Pattanayak and Tripathy, 2011). The high up-regulation of several transcripts encoding for this enzyme likely indicates that zucchini plants try to counteract aphid damage by increasing the expression of POR.

Aphid feeding spoils sieve elements. In response to this damage, plants of the Fabaceae family produce an influx of Ca^++^ that increases the volume of forisomes (proteins occurring in the sieve tubes) in order to seal the sieve tubes. As a consequence, aphids retreat their styles without phloem ingestion (Nalam et al., 2019). Although forisomes are unique to legumes, it has been proposed that a similar mechanism occurs in melon attacked by *A. gossypii* (Villada et al., 2009), thus suggesting that phloem occlusion is a mechanism to counteract aphid damage. In addition, the deposition of callose at sieve plates is induced by Ca^++^ influx into the sieve elements. In our study, several calcium-binding and calmodulin-like genes were up-regulated at the three experimental time points. This indicates that zucchini plants react to aphids by promoting the expression of calcium-related genes and subsequent Ca^++^ mobilization. The up-regulation of CUCPE_TC5771, which encodes for a sucrose synthase 6, an enzyme involved in callose deposition, further supports this hypothesis. On the other hand, it has been previously shown that mechanical damage of cell membranes after aphid puncturing leads not only to ROS formation, but also to an increase of cytosolic Ca^++^ concentration (Vincent et al., 2017) and that Ca^++^flow occurs in both compatible and incompatible plant–aphid interactions (Nalam et al., 2018). In addition, aphid saliva also contains calcium-binding proteins and can reverse phloem occlusion (Will et al., 2007). The concurrent up- and down-regulation of genes encoding for calcium-binding proteins observed in this study is consistent with a complex role of calcium in plant defense responses (Zhang et al., 2014).

Kinesin-like proteins have been associated with defense responses to tobacco mosaic virus in tomato plants (AbdelKhalek et al., 2019). Intriguingly, kinesins have shown to be involved in the transcriptional activation of genes regulating cell growth (Li et al., 2012). Our data show significant differences in the expression of a kinesin, suggesting its early activation in the control of oriented deposition of cellulose microfibrils for defense purposes; contrariwise the lack of significative up-regulation later in time may be due to aphid interference with its expression.

Salicylic acid is an essential signaling hormone for the activation of local and systemic defenses against pathogens in many plant species (Cao et al., 1998; Dempsey et al., 1999; Zhang et al., 1999; Nguyen et al., 2016). It has been proposed that phloem-feeding insects are perceived as pathogens on the basis of the similarities existing between the penetration of plant tissues by the fungal hyphae and the aphid stylets (Walling, 2000). In agreement with this view, we observed the induction of a key enzyme of the SA biosynthetic pathway (ICS1, CUCPE_TC1380) and of SA-regulated genes as PR and nudix hydrolases. The induction of SA-regulated PR genes following aphid infestation has been described in several plant species although their modes of action are not clear (Moran and Thompson, 2001; Zhu-Salzman et al., 2004; Smith and Boyko, 2007). Nudix hydrolases constitute a large family of proteins that hydrolyze nucleoside diphosphate derivates. Some of them, like *AtNUDX8*, are involved in plant immunity as positive regulator of defense (Fonseca and Dong, 2014), while other members, like *AtNUDT7*, have been identified as negative regulator of the defense response in Arabidopsis (Ge and Xia, 2008). The up-regulation of NUDX8 encoding gene (CUCPE_TC17968) at 24 hpi and the down-regulation of other nudix members (CUCPE_L17789_T_1, CUCPE_TC20679) at 48 hpi are likely a specific reaction of zucchini plant that activates, early in the infestation, a positive regulator of immunity and inactivates later the negative regulator. Our results are consistent with previous observations on the basis of which SA does not always enhance plant resistance against aphids (Moran and Thompson, 2001; Pegadaraju et al., 2005).

It has been proposed that aphid infestation induces the up-regulation of the SA-dependent pathway and reduce the expression of JA-dependent genes since aphids, in general, have been shown to be more sensitive to plant defense involving the JA signaling pathway (Kuśnierczyk et al., 2008; Giordanengo et al., 2010). In addition, previous observations showed that Arabidopsis mutants with constitutive activation of JA-signaling were more resistant to aphids than wild-type plants (Ellis et al., 2002). In agreement with this, we observed the down-regulation of several JA-related genes such as proteinase inhibitors and defensins. This can be the consequence of the up-regulation of the SA-dependent pathway or might be caused by aphid effectors (Kant et al., 2015).

Indeed, while feeding and probing, aphids convey, into the host, effector proteins that play important role in plant–aphid interactions. The down-regulation of key-genes of plant immunity, such as LRR protein kinases, phytosulfokine receptors, transcription factors, and genes associated with direct (i.e., protease inhibitors, cysteine peptidases, etc.) and indirect (i.e., terpene synthase) defenses, or some other genes above discussed, is coherent with the aphid ability to evade host defenses by secreting effectors capable of suppressing plant immune responses (Will et al., 2007; Elzinga et al., 2014; Coppola et al., 2019).

Interestingly, a variable expression of members of the cytochrome P450 family was observed. The cytochrome P450 (CYP) superfamily includes enzymes that play vital roles in promoting plant defenses *via* several biosynthetic and detoxification pathways (Ohkawa et al., 1998; Li et al., 2012). For example, CYP74 participates in the synthesis of oxylipin derivatives in the octadecanoid and jasmonate pathways (Duan et al., 2005; Brühlmann et al., 2013; Yoeun et al., 2013). A transcript related to CYP74 (CUCPE_L750_T_2) was down-regulated at 96 hpi and this was consistent with the observed down-regulation of JA-related genes. Notably, no gene related to ET biosynthesis was up-regulated in infested zucchini plants although the ET responsive transcription factor AP2/ERF (CUCPE_TC228) was up-regulated at 48 hpi. The expression of key ET-associated genes in both resistant and susceptible melon–*A. gossypii* interaction showed remarkable differences in the expression of genes in the ET signaling pathway and in downstream responses. Those genes were highly up-regulated in the resistant genotype, thus indicating that ET plays a key role in melon resistance to aphid (Anstead et al., 2010). On the other hand, a recent study showed that inhibition of ET signaling prevented aphids settling on plants (Bak et al., 2019). It appears that the aphid susceptible cultivar used in this study, although exhibiting a strong reaction, activated ineffective defense gene sets, perhaps because the aphids had conditioned its host to undermine its ability to mount effective defense responses.

### Aphid Density and Time of Plant Infestation Modify (*E*)-β-Caryophyllene Emission

Many studies have shown that plants produce VOCs in response to attack by herbivorous insects and that they can contribute to plant defenses. VOCs act as signaling molecules perceived by different trophic levels, for example, predators or parasitoids of the herbivore that are attracted toward the emitting plants (Dicke and Sabelis, 1988; Arimura et al., 2009; Dicke and Baldwin, 2010 and the references therein). Herbivore-induced VOC emissions discourage further pest attacks (Kessler and Baldwin, 2001) or serve as airborne phytohormones inducing defense responses in the non-attacked tissues of the same plant (Heil and Bueno, 2007; Coppola et al., 2018) or in neighboring plants (Baldwin et al., 2006; Coppola et al., 2017). Taken together, these observations demonstrate that induced VOC emissions are important for plant defense strategies against herbivorous insects. Several studies reported ample differences in the volatile compounds emitted from aphid-infested compared with un-infested plants (Zhu and Park, 2005; Pareja et al., 2009) although in some cases very small or no differences were observed (Turlings et al., 1998). Similarly, we found very few differences between VOCs emitted from infested and un-infested zucchini plants. In addition, we observed a slight variation in VOCs collected from control and infested plants even when the plant was infested with a much higher number of aphids. These results suggested that, at least at 48 hpi, the low emission of VOCs was not dependent on the density of aphid infestation.

Interestingly, we observed significant differences in (*E*)-β-caryophyllene between control and plants infested with low or high aphid density and for a longer time. (*E*)-β-caryophyllene is a key sesquiterpene extensively found in plants (Cai et al., 2002) produced by the activity of several terpene synthases (Köllner et al., 2008). It plays a remarkable role in plant defense, including repelling spider mites and attracting herbivore enemies above or belowground (da Camara et al., 2015). For example, in aphid-infested tomato plants (*E*)-β-caryophyllene was among the most concentrated VOCs emitted, consistently with the increased attraction of aphid parasitoids to damaged plants (Guerrieri and Digilio, 2008). Similarly, in maize, (*E*)-β-caryophyllene attracts natural enemies of herbivores (Köllner et al., 2008). Likewise, in our study, we observed that (*E*)-β-caryophyllene attracted *A. colemani*, a parasitic wasp of *A. gossypii*, which can negatively impact aphid fitness. The reduced release of (*E*)-β-caryophyllene observed at 48 hpi with five aphids is likely the result of aphid effectors that may modify several plant defense tools including the emission of VOCs, as also shown for other plant–aphid interaction (Schwartzberg et al., 2011). Coherently with this observation we registered at 48 hpi the down-regulation of a *NUDX1* (CUCPE_TC20679), a gene associated with the biosynthesis of sesquiterpenoids (Sun et al., 2020) and of a gene associated with isopentenyl diphosphate biosynthesis (CUCPE_TC9431), a compound associated with terpenoid biosynthesis (McCaskill and Croteau, 1999), that might explain the reduced release of (*E*)-β-caryophyllene observed at this time point. In addition, a strong down-regulation of a gene encoding for a terpene synthase was observed at 96 hpi suggesting a possible further reduction of (*E*)-β-caryophyllene, or other terpenes, at a low aphid density. Terpene synthases use multiple substrates and their activity could lead to important changes in several terpene products upon substrate changes like those that occur under perturbation of metabolism in stressed plants (Pazouki and Niinemets, 2016). Intriguingly, (*E*)-β-caryophyllene increased when plants were subjected to a much longer time of infestation with high aphid density. The highest amount was reached after 7 days of infestation. A possible explanation is that the increased number of aphids boosted the production of jasmonate, as previously shown (Pineda et al., 2017), that in turn increased the emission of (*E*)-β-caryophyllene (Hare, 2007).

## Conclusion

Although the interaction of zucchini “San Pasquale” with *A. gossypii* is compatible and allows aphid to settle and reproduce, plants reacted very strongly to aphid attack by reconfiguring their transcriptome. This could be interpreted as the plant trying to neutralize the pest invasion while and balancing the energy required to grow with that needed for defense. However, at least in part, changes could be due to aphid conditioning of its host plant and suppression of defense. Hundreds of genes modified their expression but, as expected for a compatible interaction, beside the lack of an R gene, the plant showed other major constrains such as (i) the late activation of immune receptors that define the first line of defense; (ii) the lack of induction of the ET regulatory pathway that plays a key role in resistance to aphids; (iii) the inability to reduce the impact of aphid effectors on its defense molecular responses. Aphids very effectively suppressed the regulation of genes encoding for undesirable compounds, such as protease inhibitors, and reduced the emission of (*E*)-β-caryophyllene at least at a low aphid density and at a short infestation time. On the other hand, this compound proved very effective in the attraction of the aphid’s natural enemy suggesting an interesting role in zucchini indirect defense. Novel genotypes able to respond to aphid attacks with the early induction of immune receptor proteins and of the ET biosynthetic pathway will more effectively counteract aphid damage.

## Data Availability Statement

The datasets presented in this study can be found in online repositories. The names of the repository/repositories and accession number(s) can be found below: Sequence Read Archive (SRA): accession number SRP136062 (PRJNA439198). Transcriptome Shotgun Assembly: accession number GGKS00000000.

## Author Contributions

AV set up the experiment, processed samples for RNA isolation, performed all bioinformatic and biochemical analyses, and drafted the early version of the manuscript. DM performed the MapMan analysis and contributed to the experimental work. MD contributed to experimental design and performed aphid infestations. MG contributed to aphid infestations. GC contributed to bioinformatic analyses. TB coordinated and supervised biochemical analysis. ND’A developed the workflow for NGS data analysis, coordinated and supervised bioinformatic work, and revised the manuscript. RR conceived the work, designed the experiment, and wrote the manuscript. All authors read and approved the final manuscript.

## Conflict of Interest

The authors declare that the research was conducted in the absence of any commercial or financial relationships that could be construed as a potential conflict of interest.
