# Supplementary material for: Zucchini Plants Alter Gene Expression and Emission of (E)-β-Caryophyllene Following Aphis gossypii Infestation
Source: Front Plant Sci. 2021 Jan 8;11:592603. doi: 10.3389/fpls.2020.592603 (PMC7820395; doi:10.3389/fpls.2020.592603)
Supplement: Supplementary file 2 [file Table_2.docx]

Supplementary Material


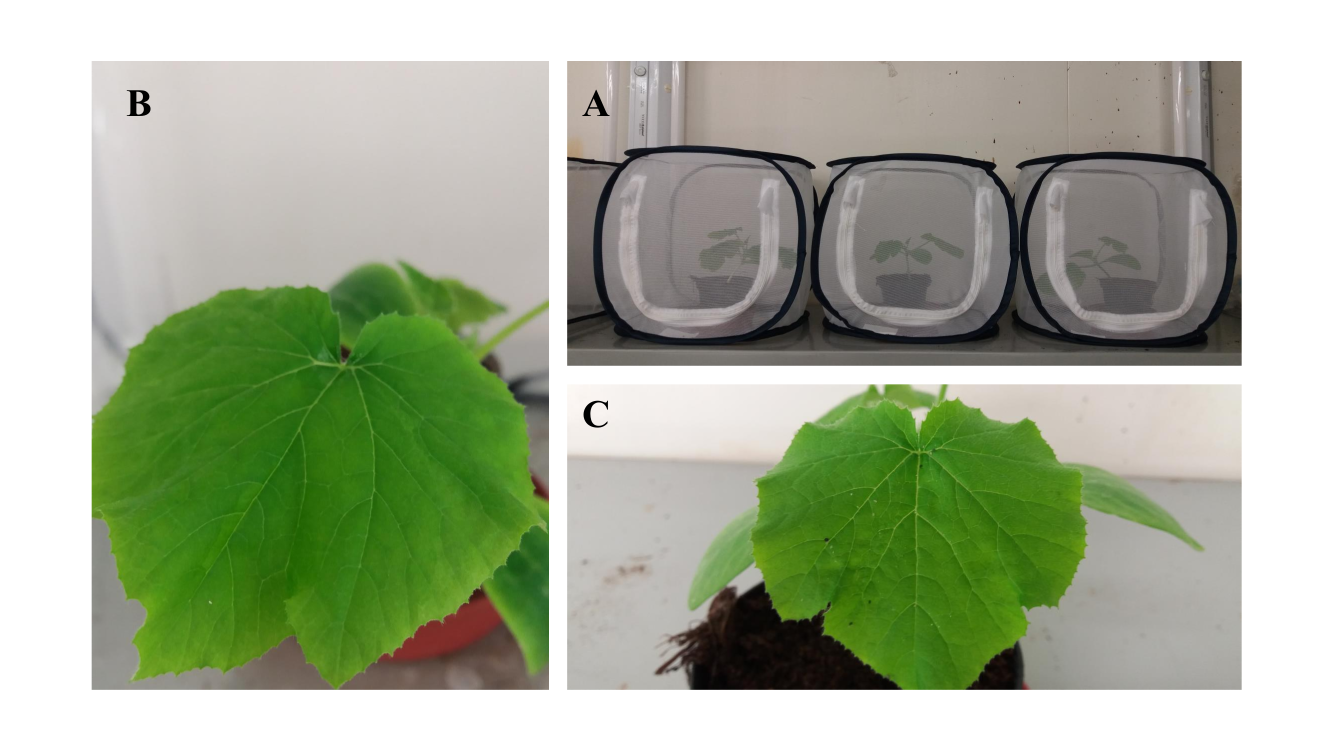


**Supplementary Figure S1.** Zucchini plants (**A**) individually arranged in insect-proof cages for the aphid infestation assay. Zoom in of zucchini leaves from (**B**) control and (**C**) aphid-infested plants at 48 hpi (hours post-infestation).


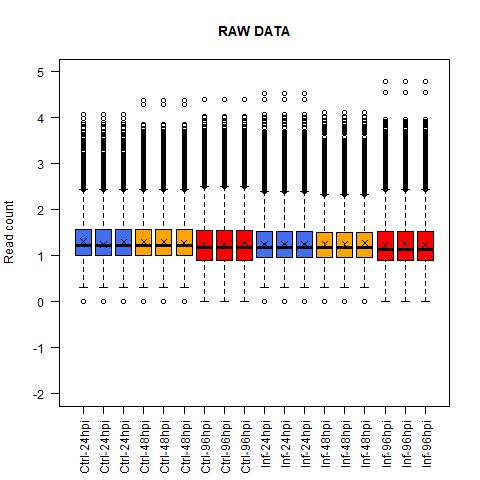

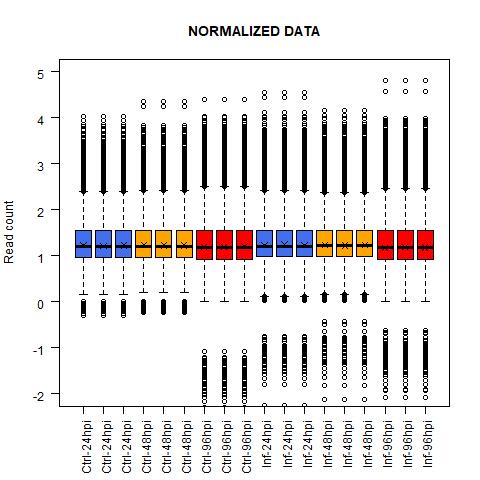


**B**

**A**

**Supplementary Figure S2.** Box plots of read counts (**A**) before and (**B)** after TMM normalization. Biological replicates referring to the same experimental time point are represented with the same colour: blue, 24 hpi; yellow, 48 hpi; red, 96 hpi. Ctrl: control samples; Inf: aphid-infested samples; hpi: hours post-infestation.

**B**

**A**


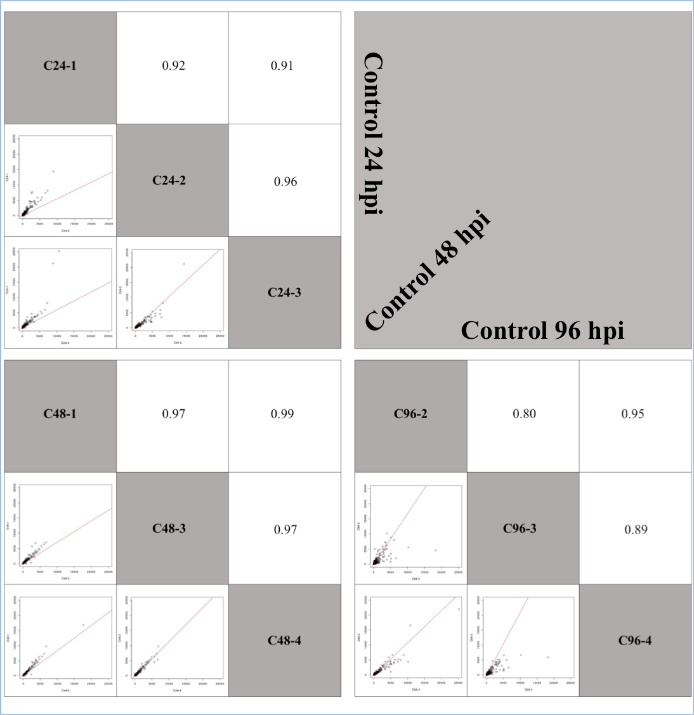

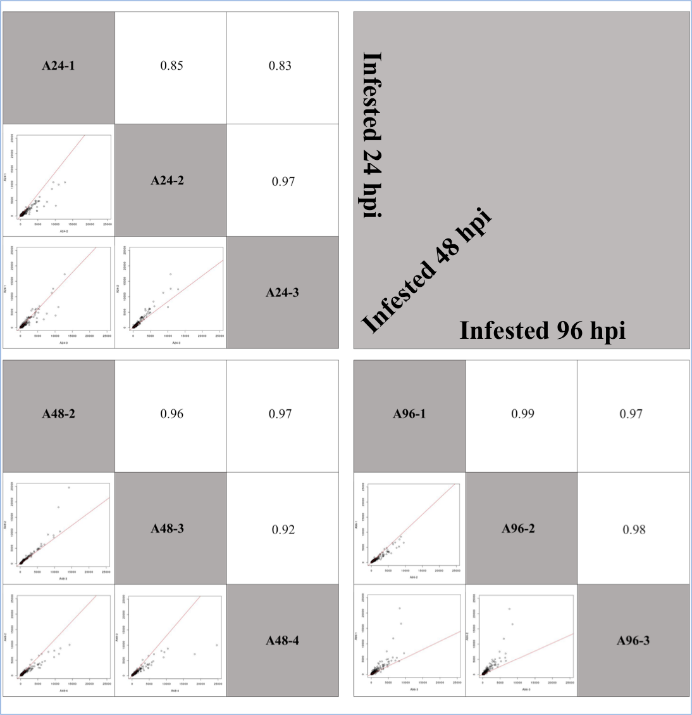


**Supplementary Figure S3**. Scatter plots and Pearson's correlation coefficient (r^2^). Relationships among RNA-Seq expression estimates in three biological replicates of control (**A**) and *Aphis gossypii* treated (**B**) samples at 24, 48 and 96 hpi (hours post-infestation).


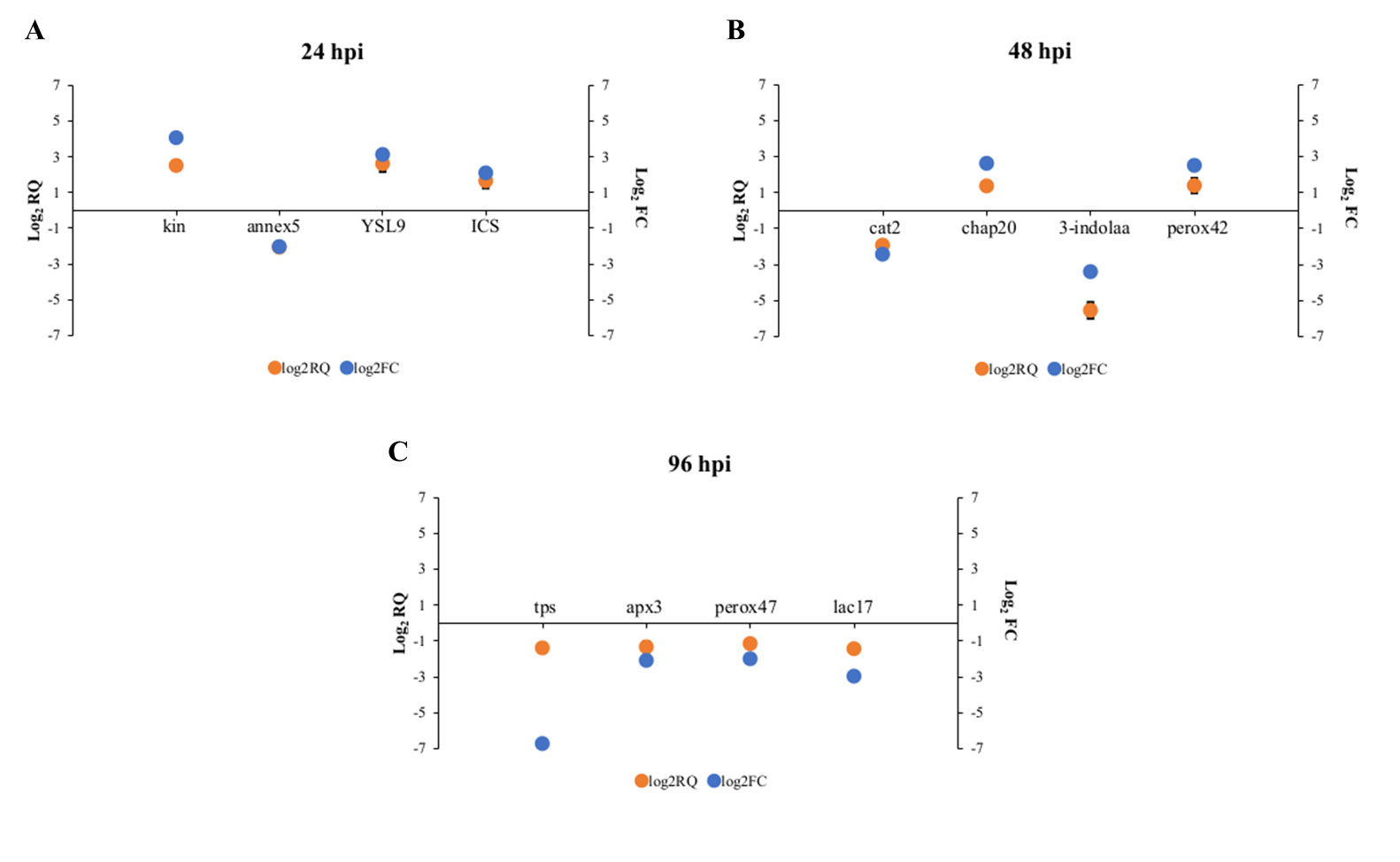


**Supplementary Figure S4**. Comparison between Log_2_ RQ (from qRT-PCR) values and Log_2_ FC (from RNA-seq) of 12 selected genes from the zucchini DEGs in plants infested by aphids at (**A**) 24, (**B**) 48 and (**C**) 96 hours post-infestation (hpi). Three biological replicates were considered for both experiments. Gene acronyms are reported in Supplementary Table S1.


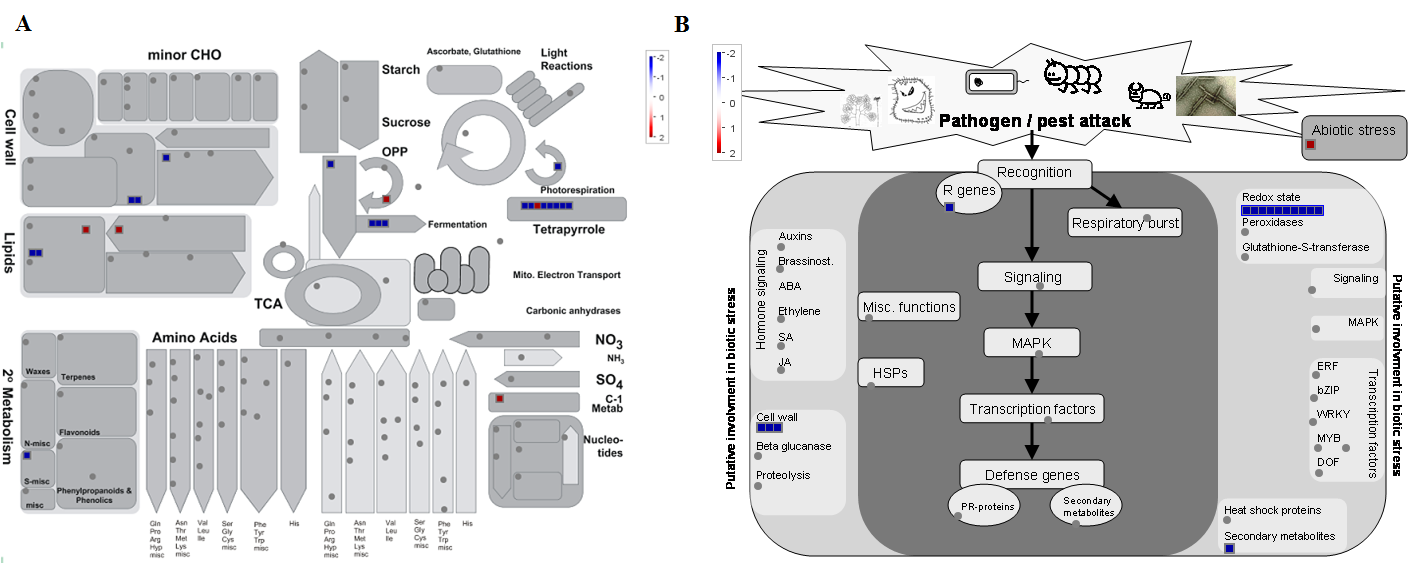


**Supplementary Figure S5**. MapMan overview of zucchini differentially expressed genes at 24 hours post-infestation by *Aphis gossypi* related to metabolism (**A**) and biotic stress (**B**). Red and blue boxes correspond to up-regulated and down-regulated genes, respectively. The grey circles represent not differentially expressed genes.


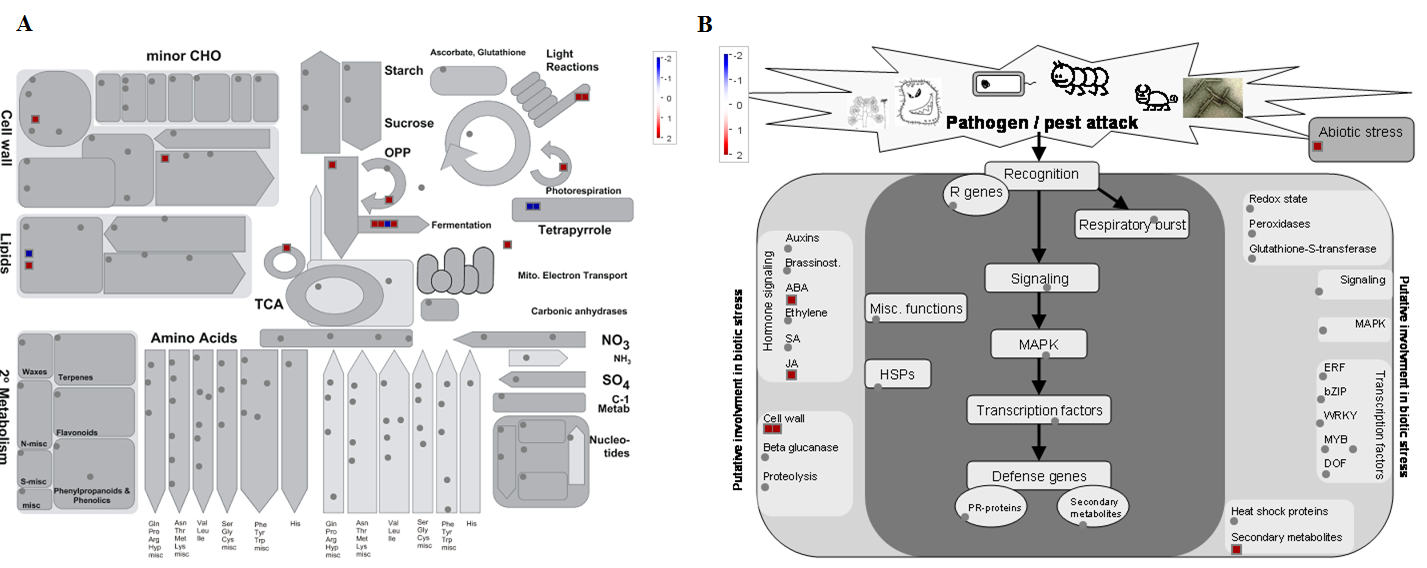


**Supplementary Figure S6**. MapMan overview of zucchini differentially expressed genes at 96 hours post-infestation by *Aphis gossypi* related to metabolism (**A**) and biotic stress (**B**). Red and blue boxes correspond to up-regulated and down-regulated genes, respectively. The grey circles represent not differentially expressed genes.


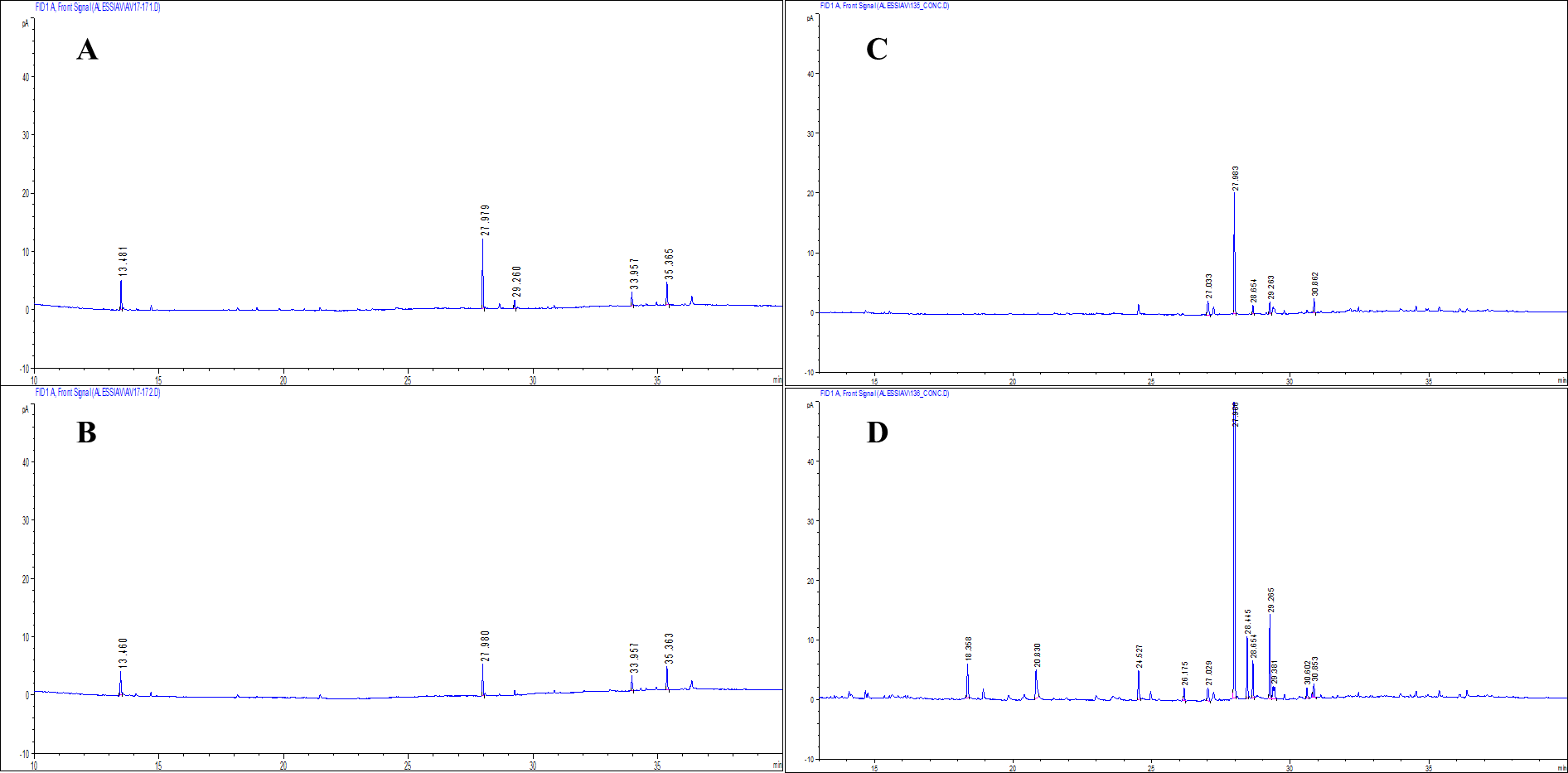


**Supplementary Figure S7.** GC analysis of VOCs collected using the Porapak polymer from (**A**) control zucchini plants and (**B**) infested by 5 *Aphis gossipii.* At 48 hours post-infestation aphids were removed from the leaves and volatile compounds were collected for the next 24 hours. The same polymer was used to collect VOCs emitted from (**C**) control zucchini plants and (**D**) infested by 300 *A. gossipii*. For samples in (**C**) and (**D**) volatile compounds were collected starting from the 4^th^ day of infestation and up to the 7^th^.

**
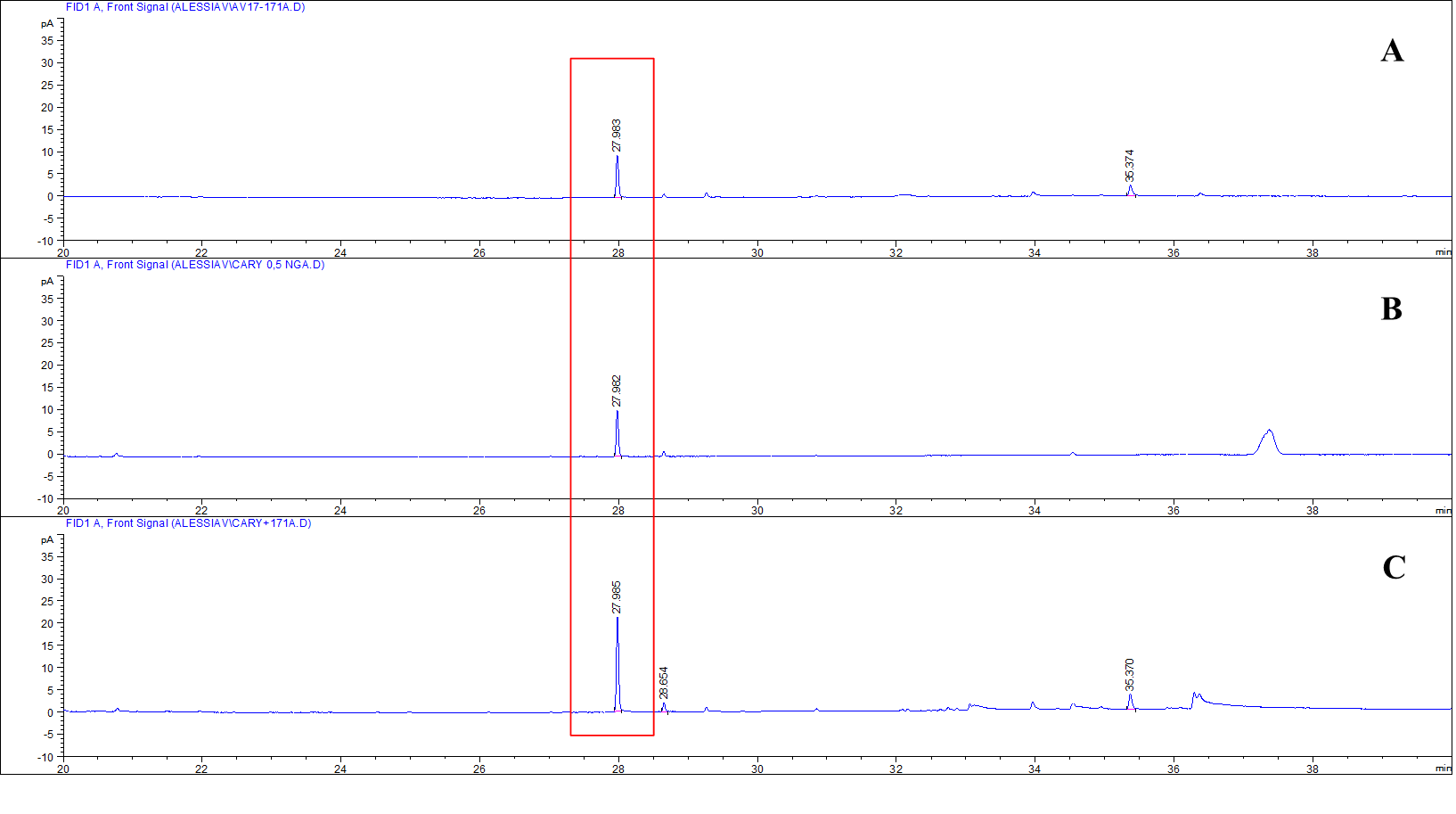
**

**Supplementary Figure S8**. Chromatograms obtained after injection on HP-1 column of (**A**) air entrainment sample from a plant infested with 5 *Aphis gossypii* for 48 h, (**B**) (*E*)-β-caryophyllene authentic standard and (**C**) co-injection of air entrainment sample and chemical standard. The (*E*)-β-caryophyllene peak is in the red box.

**Supplementary Table S1.** List of primer pairs used in qRT-PCR experiments.

**Supplementary Table S2.** Complete list of DEGs in at least one out of three comparisons. For each transcript at different time points (namely 24, 48, and 96 hours post-infestation (hpi)), it is reported the expression level estimate (Log2 Fold-change) and the statistical significance value as calculate by edgeR (FDR). Expression and statistical significance values that meet threshold criteria (-2 ≤ Log2 Fold-change ≥ 2 and FDR <0.05) are highlighted in grey. Functional annotation is obtained using the following resources: *A. thaliana* proteins (TAIR 10, https://www.arabidopsis.org/); UniProtKB/SwissProt database (http://www.uniprot.org/downloads; release 2012_02); *C. sativus* proteins (v 1.0, http://genome.jgi.doe.gov/pages/dynamicOrganismDownload.jsf?organism=Phytozome); *C. pepo* genome (v4.1, https://bioinf.comav.upv.es/downloads/zucchini/genome_v4.1/). Gene Ontologies terms, InterPro signatures and MapMan ontologies are separated by semicolon in case of multiple matches. The SOTA cluster membership is indicated for each transcript. #N/A = not annotated; BP = biological process; MF = molecular function; CC = cellular component.
